# Matrine inhibits the migratory and invasive properties of nasopharyngeal carcinoma cells

**DOI:** 10.3892/mmr.2015.3276

**Published:** 2015-01-29

**Authors:** BIN SUN, MIN XU

**Affiliations:** Department of Otolaryngology-Head and Neck Surgery, Second Hospital of Xi’an Jiaotong University, Xi’an, Shaanxi 710061, P.R. China

**Keywords:** nuclear factor-κB, nasopharyndeal carcinoma, invasion, matrix metalloproteinase

## Abstract

Matrine is a widely used Chinese herbal medicine that has historically been used in the treatment of inflammation and cancer. However, the antimetastatic effects and associated molecular mechanisms of matrine on nasopharyngeal carcinoma (NPC) remain to be elucidated. Therefore, the aims of the present study were to assess the antimetastatic effects of matrine on NPC, and identify the underlying mechanisms. Matrine inhibited the proliferation of NPC cells *in vitro* and *in vivo*. Furthermore, matrine inhibited the migration and invasion of NPC tumor cells at doses below the toxic range. Following treatment with matrine for 24 h, there was a decrease in the protein expression levels and activities of matrix metal-loproteinase (MMP)-2 and MMP-9 in NPC-039 cells. In addition, matrine markedly reduced the expression levels of p65 and p50 in the nuclei. Combined treatment of matrine with helenalin, a nuclear factor-κB (NF-κB) inhibitor resulted in a synergistic reduction in MMP-2 and MMP-9 expression levels, and the invasive capabilities of the NPC-039 cells were also reduced. In conclusion, matrine inhibits NPC cell migration and invasion by suppressing the NF-κB pathway. These results suggest that matrine may be a potential therapeutic agent for NPC.

## Introduction

Nasopharyngeal carcinoma (NPC) is a common malignancy of the head and neck. There is a high incidence of NPC in the southern regions of China, and it is common among the Inuits of Alaska (1,2). Due to its characteristic epidemiology, pathogenesis and association with the Epstein-Barr virus, NPC markedly differs from other head and neck cancers (3). In ≤75% of cases of NPC the cancer metastasizes to the neck lymph nodes, which has an adverse effect on prognosis (4). Distant metastasis, to organs including the liver, lungs and bone, is also associated with a high risk of treatment failure (5).

Metastasis is a complex process, which includes reduction of tumor cell adhesion, degradation of the extracellular matrix (ECM), enhancement of cell motility and promotion of neo-vascularization (6). Degradation of the ECM and components of the basement membrane has an important role during the metastatic process (6,7). Therefore, the actions of proteinases, such as matrix metalloproteinases (MMPs), which can degrade the ECM and components of the basement membrane, have an important role in tumor invasion and metastasis. MMP-2 and MMP-9 can degrade the majority of ECM components and are profoundly associated with the process of cancer invasion and metastasis (8,9).

Matrine (C_15_H_24_N_2_O) is derived from the *Sophora* plant genus and has historically been used in traditional Chinese medicine to treat inflammation (10). Matrine has been shown to produce a wide range of pharmacological effects and has been used to treat various diseases, including viral hepatitis, neuropathic pain and isoproterenol-induced cardiotoxicity (11–13). No obvious toxicity or side effects of matrine have been reported. Matrine also exhibits an anticancer effect, on malignancies including gastric cancer, rhabdomyosarcoma, acute myeloid leukemia and breast cancer (14–17). However, to the best of our knowledge, there are currently no published studies regarding the antimetastatic effects of matrine on NPC. The present study aimed to investigate the effects of matrine against NPC cell invasion and metastasis, and identify the underlying mechanisms.

## Materials and methods

### Ethics statement

Male BALB/c nude mice (age, 4 weeks) were supplied by the Experimental Animal Center of Xi’an Jiaotong University (Xi’an, China). The present study was conducted according to the recommended guidelines for the Care and Use of Laboratory Animals, issued by the Chinese Council on Animal Research. The protocol was approved by the Ethics Committee of Xi’an Jiaotong University.

### Reagents

Matrine was obtained from Sigma-Aldrich (St. Louis, MO, USA) and was dissolved in dimethyl sulfoxide (DMSO) for cell culture. Fetal bovine serum (FBS), penicillin and streptomycin were all purchased from Gibco Life Technologies (Carlsbad, CA, USA). Helenalin was purchased from Sigma-Aldrich. Mouse monoclonal anti-nuclear factor-κ (NF-κB) p50 (sc-271908), mouse monoclonal anti-NF-κ p65 (sc-71676), mouse monoclonal anti-β-actin (sc-376421) and rabbit polyclonal anti-histone H1 (sc-67324) were purchased from Santa Cruz Biotechnology, Inc. (Dallas, TX, USA). Rabbit polyclonal anti-MMP-2 (#4002) and rabbit polyclonal anti-MMP-9 (#2270) antibodies were purchased from Cell Signaling Technologies (Danvers, MA, USA).

### Cell culture

The NPC-039 cells were obtained from Academia Sinica (Taipei, China) and were was cultured in Dulbecco’s modified Eagle’s medium (DMEM; Sigma-Aldrich) supplemented with 10% FBS. The poorly differentiated human CNE-2Z NPC cell line was obatined from Zhongshan University (Guangzhou, China) and cultured in RPMI-1640 medium (HyClone Laboratories, Inc., Logan, UT, USA) supplemented with 5% FBS and 100 units penicillin/streptomycin. All of the cells were cultured at 37°C in a humidified incubator containing 5% CO_2_.

### Assessment of cell viability

Cell viability was determined using a colorimetric 3-(4, 5-dimethylthiazol-2-yl) 2, 5-diphenyltetrazolium bromide (MTT) assay, according to previously described methods (6). Briefly, the cells were plated in 96-well culture plates (2×10^4^/well) and treated with serial concentrations (0, 12.5, 25, 50, 100 and 200 *μ*g/ml) of matrine for 24 h. Following incubation, the cells were washed twice with phosphate-buffered saline (PBS) and incubated with 5 mg/ml MTT (Sigma-Aldrich) for 4 h. The living cells absorbed the reagent and subsequently produced insoluble blue formazan. Following the incubation, the cells were washed again with PBS and solubilized using DMSO. The optical density of the cells was measured at 550 nm using an enzyme-linked immunosorbent assay plate reader (DR-200Bs; Bio-Rad Laboratories, Hercules, CA, USA).

### Invasion assays in vitro

Prior to cell seeding, a 24-well cell culture invasion chamber (Corning Inc., Tewksbury, MA, USA) was coated with 8.0 *μ*m Matrigel™ (Becton Dickinson, Bedford, MA, USA), and the CNE-2Z cells were pretreated with 0, 12.5, 25 and 50 *μ*g/ml matrine, or helenalin (5 *μ*M), for 24 h. The cells were then seeded in 200 *μ*l serum-free medium and placed in the upper chambers at 2×10^5^ cells, normal growth medium was placed in the bottom chambers. Following a 24 h incubation, the cells on the upper surface of the membrane were removed using cotton swabs, and the cells on the bottom side of the filter were fixed in 90% ethyl alcohol (Xi’an Chemical Reagent Factory, Xi’an, China), stained with Crystal Violet (Sigma-Aldrich) and counted under an Olympus-CX31 microscope (Olympus Corp., Tokyo, Japan). Spontaneous migration in DMSO was designated as the control. The rate of invasion was expressed as a percentage of the control.

### Zymography

MMP-2 and MMP-9 activity in the samples was studies using zymography. In brief, the cells were treated with different concentrations (0, 12.5, 25 and 50 *μ*g/ml) of matrine at 37°C for 24 h. The conditioned media were collected from all of the cells and lysated. The protein concentration was then measured and the final 10 *μ*l sample was subjected to electrophoresis using an 8% SDS-PAGE gel co-polymerized with 0.1% gelatin. Following electrophoresis, the gels were incubated in 2.5% Triton X-100 solution (Sigma-Aldrich) at room temperature for 1 h, and then incubated in reaction buffer (10 mM CaCl_2_, 40 mM Tris-HCl and 0.01% NaN_3_, pH 8.0) overnight at 37°C. The gels were stained with 0.1% Coomassie brilliant blue R-250 (Sigma-Aldrich) and destained with 30% methanol and 10% acetic acid. Gelatinolytic activities were detected as unstained bands against the background of Coomasie-stained gelatin. The intensities of the bands on the gels were determined using an image analysis system (Quantity One v4.62; Bio-Rad Laboratories).

### Western blot analysis

Following treatment of the NC-039 cells with different concentrations of matrine (0, 12.5, 25 and 50 *μ*g/ml) or helenalin (5 *μ*M), 1×10^6^ cells were suspended in 200 *μ*l lysis buffer (40 mmol/l Tris-HCl, 1 mmol/l EDTA, 150 mmol/l KCl, 100 mmol/l NaVO_3_, 1% Triton X-100 and 1 mmol/l PMSF, pH 7.5). Nuclear lysates from the cultured NPC-039 cells were harvested using the NucBuster™ Protein Extraction kit (Novagen^®^; Merck KGaA, Darmstadt, Germany), according to the manufacturer’s instructions. The proteins (60 *μ*g) were separated by 10% SDS-PAGE and transferred onto polyvinylidene fluoride membranes (Millipore Corp., Billerica, MA, USA). The membranes were subsequently blocked in non-fat milk [5% in Tris-buffered saline with Tween^®^-20 (TBST) buffer] at 37°C for 1 h, in order to block non-specific binding. The membranes were then incubated overnight with polyclonal antibodies against p50, p65, MMP-2, MMP-9, β-actin and Histone H1, in TBST containing 5% non-fat milk, at 4°C. The membranes were subsequently incubated with a horseradish peroxidase-conjugated goat anti-mouse (EK010) or anti-rabbit (EK020) immunoglobulin G (Zhuangzhi Bio, Xi’an, China), for 1 h at room temperature. The bands were visualized using an Enhanced Chemiluminescence kit (ECL Plus; GE Healthcare Europe GmbH, Freiburg, Germany) and exposed by autoradiography (GelDocXR+; Bio-Rad Laboratories). A densitometric analysis was conducted using ImageJ software (version 1.42q; National Institute of Health, Bethesda, MD, USA) and the results are expressed as arbitrary units (a.u.).

### Animal and tumor xenograft assays

*In vivo* tumorigenicity was achieved as described by previous methods (18). Briefly, suspensions of NPC-039 tumor cells (5×10^5^ viable cells/mouse) were implanted into the right flank region of the BALB/c nude mice. Forty-eight hours after the injection (day 1), the mice were randomly divided into two groups (n=5/group). The animals were pair matched, in order to ensure that the median tumor volume for each group was similar. The treatment group received matrine (60 mg/kg per day) by intragastric administration, and the control group received an equal volume of saline. The tumor volumes were measured twice weekly using calipers, and the volumes (cm^3^) were calculated according to the following standard formula: (length × width^2^)/2. After three weeks of drug administration, the mice were sacrificed by cervical dislocation, and the tumors were harvested and weighed. The experimental protocols involving mice in the present study were evaluated and approved by the Animal Care and Use Committee of the Medical School of Xi’an Jiaotong University.

### Wound healing assays

Wound healing assays were performed on NPC-039 cells. In brief, NPC-039 cells were seeded into a six-well plate and cultured to 60–70% confluency in medium containing 10% FBS. Cell monolayers were wounded using a plastic tip (1 mm) that touched the plate as described previously (6). NPC-039 cells were then incubated in serum-containing medium (2% serum) with oxymatrine (0, 12.5, 25 and 50 μg/ml) for 24 h. Images were captured at 0 and 24 h following the addition of oxymatrine. The migration distance of the cells was measured under an Olympus-CX31 microscope (Olympus Corp.).

### Statistical analysis

The data are expressed as the mean ± standard deviation. Statistical analyses were conducted using SPSS version 16.0 software (SPSS Inc., Chicago, IL, USA), to evaluate statistical differences. Student’s t-test was used for comparisons between two groups, and a one-way or two-way analysis of variance was used to analyze statistical differences between the groups under different conditions. P<0.05 was considered to indicate a statistically significant difference. All of the statistical tests were two sided. A correlation analysis by Z test was also performed.

## Results

### Matrine inhibits the proliferation of NPC cells in vitro

**Figure 1:**
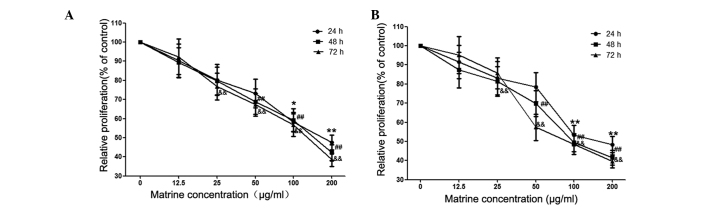
Cellular viability of NPC-039 and CNE-2Z nasopharyngeal carcinoma cells treated with matrine. (A) NPC-039 and (B) CNE-2Z cells were treated with matrine, and incubated for 24, 48 and 72 h, after which the cell viability was measured using an 3-(4, 5-dimethylthiazol-2-yl) 2, 5-diphenyltetrazolium bromide assay. The data represent the mean ± standard deviation of three independent experiments, each performed in triplicate. ^*^P<0.05 and ^**^P<0.01, compared with the untreated control group at 24 h; ^#^P<0.05 and ^##^P<0.01, compared with the control group at 48 h; ^&^P<0.05 and ^&^P<0.01, compared with the control group at 72 h.

Matrine reduced the viability of the two human NPC cell lines, in a dose-dependent manner, following treatment with 0–200 *μ*g/ml matrine for 24, 48 and 72 h (NPC-039, Fig. 1A; CNE-2Z, Fig. 1B). At concentrations <50 *μ*g/ml (24 h), the antiproliferative effects of matrine were not obvious; therefore, a concentration range lower than this was chosen for use in all subsequent experiments regarding the anti-metastatic effects of matrine.

### Matrine inhibits the migration and invasion of NPC-039 cells

**Figure 2:**
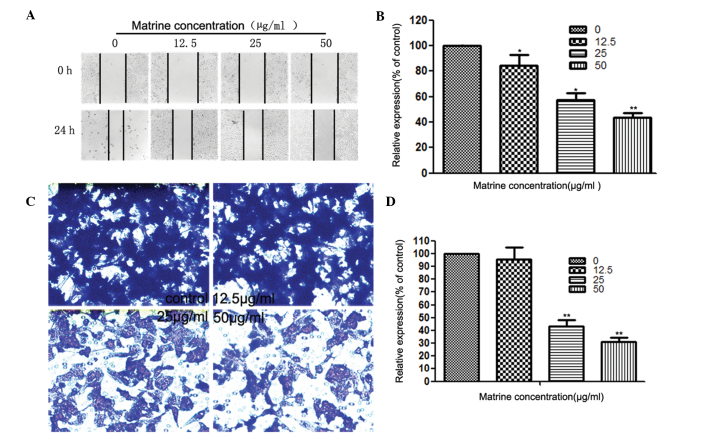
Effects of matrine on the *in vitro* invasion of NPC-039 nasopharyngeal carcinoma cells. (A) NPC-039 cells underwent a migration assay. Cells were cultured in media supplemented with 2% fetal bovine serum (FBS) and treated with various concentrations of matrine (0, 12.5, 25 and 50 *μ*g/ml). Images were captured at 0 and 24 h. (B) Rate of migration was expressed as a percentage of the control (0 *μ*g/ml). (C) NPC-039 cells underwent an invasion assay. Cells pre-incubated with various concentrations of matrine (0, 12.5, 25 and 50 *μ*g/ml) were plated onto the upper wells of a chamber. FBS (10%) was added to the bottom wells for 24 h, to induce cell invasion. Following a 24 h incubation, the cells on the bottom side of the filter were fixed, stained in crystal violet and measured. Spontaneous migration in dimethyl sulfoxide was designated as control (magnification, ×100). (D) The rate of invasion was expressed as a percentage of the control (0 *μ*g/ml). The data represent the mean ± standard deviation of three independent experiments. ^*^P<0.05 and ^**^P<0.01, compared with the control group.

As shown in Fig. 2A, the movement of NPC-039 cells was significantly reduced in response to treatment with matrine; the rate of inhibition was ~15.79, 42.86 and 56.68.1% at 24 h with 12.5, 25 and 50 *μ*g/ml matrine, respectively (Fig. 2B). The effects of matrine on the invasiveness of NPC-039 cells treated with 0, 12.5, 25 and 50 *μ*g/ml matrine for 24 h are shown in Fig. 2C. Matrine significantly reduced the invasiveness of the NPC-039 cells. Similar antimetastatic effects of matrine were observed in the CNE-2Z cells (data not shown). Quantification indicated that the invasiveness of the NPC-039 cells was reduced by 4.71, 56.75 and 68.77% in response to treatment with 12.5, 25 and 50 *μ*g/ml of matrine (Fig. 2D), respectively.

### Matrine inhibits the protein expression and activity of MMP-2 and MMP-9 in NPC-039 cells

**Figure 3:**
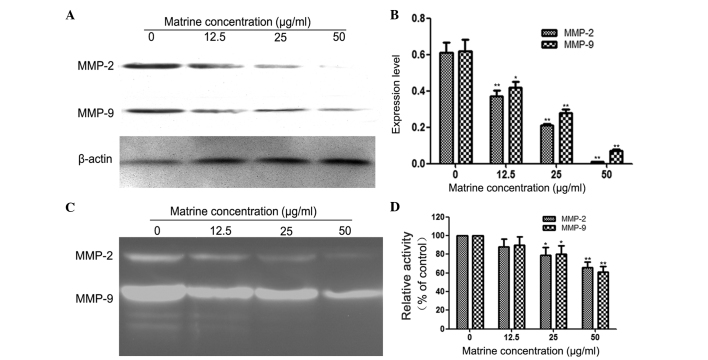
Matrine suppresses the expression of matrix metalloproteinase (MMP)-2 and MMP-9 in NPC-039 nasopharyngeal carcinoma cells. (A) NPC-039 cells were treated with matrine (0, 12.5, 25 and 50 *μ*g/ml) for 24 h and then subjected to western blotting, to analyze the protein expression levels of MMP-2 and MMP-9. (B) Quantification of the protein expression levels of MMP-2 and MMP-9 in NPC-039 cells. (C) Effects of matrine on the acivities of MMP-2 and MMP-9. (D) Quantification of the activities of MMP-2 and MMP-9 in NPC-039 cells. The data represents the mean ± standard deviation of three independent experiments, performed in triplicate. ^*^P<0.05 and ^**^P<0.01, compared with the control group.

MMP-2 and MMP-9 have important roles in the invasion of cancer cells (6). The present study analyzed the effects of matrine on the protein expression levels and the activity of MMP-2 and MMP-9. Matrine significantly reduced the protein expression levels (Fig. 3A and B) and the relative activity of MMP-2 and MMP-9 (Fig. 3C and D).

### Matrine inhibits the nuclear translocation of p50 and p65 in NPC-039 cells, and the effects of helenalin and matrine on cell invasion and MMP-2 and MMP-9 expression in NPC-039 cells

**Figure 4:**
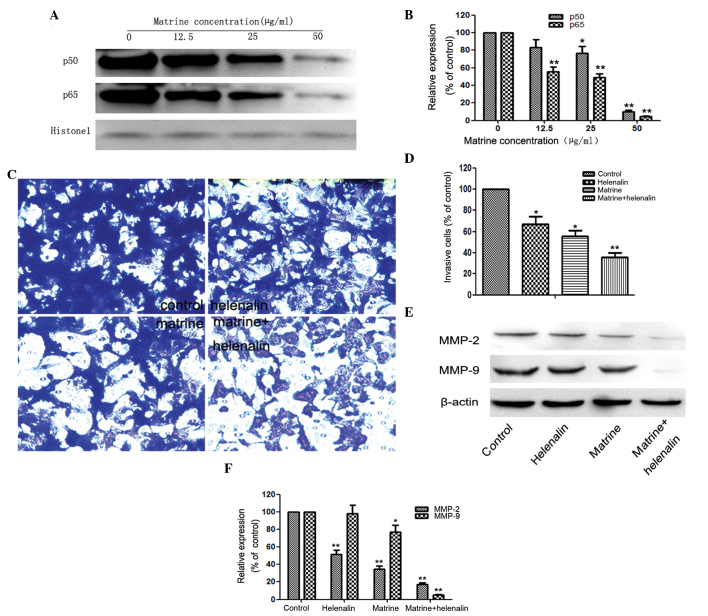
Expression levels of nuclear factor (NF)-κB-related proteins following treatment with matrine, and the effects of the NF-κB inhibitor helenalin and matrine on cell invasion and matrix metaloproteinase (MMP)-2 and MMP-9 protein expression in NPC-039 nasopharyngeal carcioma cells. (A) Inhibitory effects of matrine on p50 and p65 nuclear translocation. Cells were pretreated with matrine for 12 h. Nuclear and whole cell lysate proteins were prepared and analyzed by western blotting, with Histone H1 serving as a loading control. (B) Quantification of p50 and p65 nuclear translocation. (C) Cells were pretreated with helenalin (5 *μ*M) for 30 min and then incubated in the presence or absence of matrine (25 *μ*g/ml) for 24 h. Cellular invasiveness was measured using a Boyden chamber invasion assay and cells were stained with crystal violet. Control group, cells were left untreated (magnification, ×100). (D) The rate of invasion was expressed as a percentage of control. (E and F) NPC-039 cells were treated and then subjected to western blotting to analyze the protein expression levels of MMP-2 and MMP-9. The data represents the mean ± standard deviation of three independent experiments, performed in triplicate. ^*^P<0.05 and ^**^P<0.01, compared with the control group.

NF-κB has an important role in controlling tumor cell migration; therefore, the present study hypothesized that the reduction in NPC cell invasion may be a result of downregulation of NF-κB. The protein expression levels of p65 and p50 in the nuclei of NPC-039 cells were markedly downregulated following treatment with matrine (Fig. 4A and B). NF-κB has previously been reported as a downstream target of matrine in numerous types of cells, which results in the upregulation of MMP-2 and MMP-9 expression (19–21). The results of the present study indicate that combined treatment of an NF-κB inhibitor, henenalin, and matrine, results in a synergistic reduction in cell invation (Fig. 4C and D) as well as MMP-2 and MMP-9 expression (Fig. 4E and F).

### In vivo inhibition of NPC tumor growth by matrine

**Figure 5:**
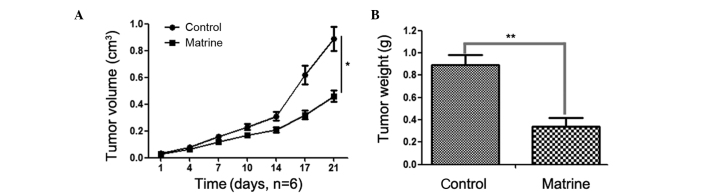
*In vivo* growth inhibition of implanted NPC-039 nasopharyngeal carcinoma cells in nude Balb/c mice by matrine. NPC-039 cells were implanted subcutaneously into nude mice. Mice were treated with or without matrine by intraperitoneal injection (60 mg/kg/day). (A) The relative tumor volume and (B) final tumor weight of control and matrine-treated mice impanted with NPC-039 cells. ^*^P<0.05 and ^**^P<0.01, compared with vehicle treatment.

To evaluate the *in vivo* effects of matrine on tumor growth, NPC-039 cells were xenografted into nude mice, as described by previous methods (18). The time course of NPC-039 xenograft growth, with and without matrine treatment, is shown in Fig. 5A. A significant inhibition of tumor growth was observed in response to treatment with matrine. In the matrine-treated (60 mg/kg/day) mice, 21 days after cell implantation the NPC-039 xenograft volumes were significantly inhibited. At the end of the experiment, the xenograft tumors were harvested and weighed. Matrine was found to significantly decrease the solid tumor mass, as compared with the control group (Fig. 5B).

## Discussion

Metastasis is currently considered to be the main obstacle in the clinical management of NPC. Prevention, prediction and inhibition of NPC metastasis is critical to further improve the survival rate. The present study demonstrated that matrine was a strong metastatic inhibitor for NPC. Matrine has previously been proposed as a potential drug for certain types of tumor (22,23). However, the antimetastatic effects and related mechanisms in NPC cells remained unclear. In the present study, matrine suppressed the migratory and invasive ability of NPC cells, at doses below its toxic range. This is the first scientific report, to the best of our knowledge, regarding the antimetastatic effects of matrine on NPC.

To determine the effects of matrine on the motility, migration and invasiveness of NPC cells, a wound healing migration assay and a Boyden chamber invasion assay were employed, respectively. Matrine significantly inhibited the migration and invasion of the NPC cells. Metastasis is a complex process, during which degradation of components of the ECM is a key step (6); since the loss of ECM integrity allows the cancer cells to invade the blood or lymphatic system, and spread to other tissues and organs. MMP-2 and MMP-9 have important roles in the degradation of ECM (24–26). Previous studies have shown that matrine can reduce the expression of MMP-2 and MMP-9 in numerous types of cells (19–21,27). Therefore, the present study investigated the effects of matrine on the expression levels of MMP-2 and MMP-9 in NPC-039 cells, and demonstrated that matrine could significantly reduce the expression and activity of MMP-2 and MMP-9. These results indicate that matrine may inhibit the invasion of NPC-039 cells by regulating the expression and activity of MMP-2 and MMP-9.

NF-κB is able to upregulate MMP-2 and MMP-9 expression (19–21), and has previously been reported as a downstream target of matrine in numerous cell types (28–31). In the present study, matrine was shown to significantly increase the expression levels of p65 and p50 within the nuclei of NPC-039 cells. The results of the present study also indicated that cellular motility inhibited by matrine may be enhanced by blocking the NF-κB pathway, thus providing further evidence that NF-κB is a key signaling pathway by which matrine regulates the migratory and invasive abilities of NPC cells.

I*n vivo* analyses of the present study demonstrated that matrine decreased the growth of NPC-039 cell tumor xenografts in nude mice by inhibiting cell proliferation. In conclusion, the present study demonstrated the inhibitory effects of matrine on the migration and invasion of NPC cells. Furthermore, the decreased expression levels of MMP-2 and MMP-9, induced by matrine, may be attributed to inhibition of the NF-κB pathway. These results reveal a novel potential therapeutic application of matrine in antimetastatic therapy for NPC.

## References

[1] Albeck H, Bentzen J, Ockelmann HH, Nielsen NH, Bretlau P, Hansen HS (1993). Familial clusters of nasopharyngeal carcinoma and salivary gland carcinomas in Greenland natives. Cancer.

[2] Liu A, Huang W, Zeng G, Ma X, Zhou X, Wang Y, Ouyang C, Cheng A (2014). Expression of the Annexin A1 gene is associated with suppression of growth, invasion and metastasis of nasopharyngeal carcinoma. Mol Med Rep.

[3] Wei WI, Sham JS (2005). Nasopharyngeal carcinoma. Lancet.

[4] Chua DT, Sham JS, Wei WI, Ho WK, Au GK (2001). The predictive value of the 1997 american joint committee on cancer stage classification in determining failure patterns in nasopharyngeal carcinoma. Cancer.

[5] Cheng SH, Jian JJ, Tsai SY (1998). Prognostic features and treatment outcome in locoregionally advanced nasopharyngeal carcinoma following concurrent chemotherapy and radiotherapy. Int J Radiat Oncol Biol Phys.

[6] Chen K, Zhang S, Ji Y (2013). Baicalein inhibits the invasion and metastatic capabilities of hepatocellular carcinoma cells via down-regulation of the ERK pathway. PLoS One.

[7] Raghu H, Sodadasu PK, Malla RR, Gondi CS, Estes N, Rao JS (2010). Localization of uPAR and MMP-9 in lipid rafts is critical for migration, invasion and angiogenesis in human breast cancer cells. BMC Cancer.

[8] Bjorklund M, Koivunen E (2005). Gelatinase-mediated migration and invasion of cancer cells. Biochim Biophys Acta.

[9] Yang SF, Hsieh YS, Lin CL (2005). Increased plasma levels of urokinase plasminogen activator and matrix metalloproteinase-9 in nonsmall cell lung cancer patients. Clin Chim Acta.

[10] Li Y, Wang B, Zhou C, Bi Y (2007). Matrine induces apoptosis in angiotensin II-stimulated hyperplasia of cardiac fibroblasts: effects on Bcl-2/Bax expression and caspase-3 activation. Basic Clin Pharmacol Toxicol.

[11] Long Y, Lin XT, Zeng KL, Zhang L (2004). Efficacy of intramuscular matrine in the treatment of chronic hepatitis B. Hepatobiliary Pancreat Dis Int.

[12] Haiyan W, Yuxiang L, Linglu D (2013). Antinociceptive effects of matrine on neuropathic pain induced by chronic constriction injury. Pharm Biol.

[13] Li X, Zhou R, Zheng P (2010). Cardioprotective effect of matrine on isoproterenol-induced cardiotoxicity in rats. J Pharm Pharmacol.

[14] Li Y, Zhang J, Ma H (2013). Protective role of autophagy in matrine-induced gastric cancer cell death. Int J Oncol.

[15] Guo L, Xue TY, Xu W, Gao JZ (2012). Effects of matrine on the proliferation and apoptosis of human rhabdomyosarcoma RD cells. Zhongguo Dang Dai Er Ke Za Zhi.

[16] Zhang S, Zhang Y, Zhuang Y (2012). Matrine induces apoptosis in human acute myeloid leukemia cells via the mitochondrial pathway and Akt inactivation. PLoS One.

[17] Li LQ, Li XL, Wang L (2012). Matrine inhibits breast cancer growth via miR-21/PTEN/Akt pathway in MCF-7 cells. Cell Physiol Biochem.

[18] Li M, Su BS, Chang LH (2014). Oxymatrine induces apoptosis in human cervical cancer cells through guanine nucleotide depletion. Anticancer Drugs.

[19] Yu HB, Zhang HF, Li DY, Zhang X, Xue HZ, Zhao SH (2011). Matrine inhibits matrix metalloproteinase-9 expression and invasion of human hepatocellular carcinoma cells. J Asian Nat Prod Res.

[20] Yu P, Liu Q, Liu K, Yagasaki K, Wu E, Zhang G (2009). Matrine suppresses breast cancer cell proliferation and invasion via VEGF-Akt-NF-kappaB signaling. Cytotechnology.

[21] Zhang W, Dai BT, Xu YH (2008). Effects of matrine on invasion and metastasis of leukemia cell line jurkat. Zhongguo Zhong Xi Yi Jie He Za Zhi.

[22] Song S, Zhu S, Zhang Z, Mo Z, Ke Q, Luo Z (2013). A study on the inhibitory effect of matrine on gastric cancer SGC-7901 cells. Afr J Tradit Complement Altern Med.

[23] Chang C, Liu SP, Fang CH (2013). Effects of matrine on the proliferation of HT29 human colon cancer cells and its antitumor mechanism. Oncol Lett.

[24] Gialeli C, Theocharis AD, Karamanos NK (2011). Roles of matrix metalloproteinases in cancer progression and their pharmacological targeting. FEBS J.

[25] Yeh CB, Hsieh MJ, Hsieh YH, Chien MH, Chiou HL, Yang SF (2012). Antimetastatic effects of norcantharidin on hepatocellular carcinoma by transcriptional inhibition of MMP-9 through modulation of NF-kB activity. PLoS One.

[26] Yeh CB, Hsieh MJ, Hsieh YS (2012). Terminalia catappa exerts antimetastatic effects on hepatocellular carcinoma through transcriptional inhibition of matrix metalloproteinase-9 by modulating NF-kappaB and AP-1 activity. Evid Based Complement Alternat Med.

[27] Zhang YJ, Xiang MX, San J, Cheng G, Wang SS (2006). Effect of matrine and carvedilol on collagen and MMPs activity of hypertrophy myocardium induced by pressure overload. J Zhejiang Univ Sci B.

[28] Gao H, He S, Tang WX, Wang J (2007). Inhibition of NF-kappa B activity enhanced apoptosis induced by matrine in hepatocellular carcinoma cells. Zhonghua Gan Zang Bing Za Zhi.

[29] Hu H, Wang S, Zhang C (2010). Synthesis and in vitro inhibitory activity of matrine derivatives towards pro-inflammatory cytokines. Bioorg Med Chem Lett.

[30] Zhang B, Liu ZY, Li YY (2011). Antiinflammatory effects of matrine in LPS-induced acute lung injury in mice. Eur J Pharm Sci.

[31] Xu M, Yang L, Hong LZ, Zhao XY, Zhang HL (2012). Direct protection of neurons and astrocytes by matrine via inhibition of the NF-kappaB signaling pathway contributes to neuroprotection against focal cerebral ischemia. Brain Res.

